# Supplementary material for: HIVprotI: an integrated web based platform for prediction and design of HIV proteins inhibitors
Source: J Cheminform. 2018 Mar 9;10:12. doi: 10.1186/s13321-018-0266-y (PMC5845081; doi:10.1186/s13321-018-0266-y)
Supplement: Supplementary file 2 — Additional file 2. Source code of HIVProtI web server. [file 13321_2018_266_MOESM2_ESM.zip › HIVprotI_Source-code/hivprotI/weka-3-6-6/doc/weka/clusterers/FilteredClusterer.html]

FilteredClusterer


---


|  |  |  |
| --- | --- | --- |
| **Overview** \| **Package** \| **Class** \| **Tree** \| **Deprecated** \| **Index** \| **Help** \| **Weka's home** | |  |
| **PREV CLASS**   **NEXT CLASS** | **FRAMES**    **NO FRAMES**     **All Classes** |  |
| SUMMARY: NESTED \| FIELD \| CONSTR \| METHOD | DETAIL: FIELD \| CONSTR \| METHOD |  |


---


## weka.clusterers Class FilteredClusterer

```
java.lang.Object
  weka.clusterers.AbstractClusterer
      weka.clusterers.SingleClustererEnhancer
          weka.clusterers.FilteredClusterer
```

**All Implemented Interfaces:**: java.io.Serializable, java.lang.Cloneable, Clusterer, CapabilitiesHandler, OptionHandler, RevisionHandler

---

``` public class FilteredClusterer extends SingleClustererEnhancer ```

Class for running an arbitrary clusterer on data that has been passed through an arbitrary filter. Like the clusterer, the structure of the filter is based exclusively on the training data and test instances will be processed by the filter without changing their structure.


Valid options are:

```
 -F <filter specification>
  Full class name of filter to use, followed
  by filter options.
  eg: "weka.filters.unsupervised.attribute.Remove -V -R 1,2"
 (default: weka.filters.AllFilter)
```

```
 -W
  Full name of base clusterer.
  (default: weka.clusterers.SimpleKMeans)
```

```
 Options specific to clusterer weka.clusterers.SimpleKMeans:
```

```
 -N <num>
  number of clusters.
  (default 2).
```

```
 -V
  Display std. deviations for centroids.
```

```
 -M
  Replace missing values with mean/mode.
```

```
 -S <num>
  Random number seed.
  (default 10)
```

Based on code from the FilteredClassifier by Len Trigg.

**Version:**
:   $Revision: 5538 $

**Author:**
:   Len Trigg (trigg@cs.waikato.ac.nz), FracPete (fracpete at waikato dot ac dot nz)

**See Also:**: `FilteredClassifier`, Serialized Form

---

**Constructor Summary**

`FilteredClusterer()`
  
          Default constructor.


| **Method Summary** | |
| --- | --- |
| `void` | `buildClusterer(Instances data)`             Build the clusterer on the filtered data. |
| `double[]` | `distributionForInstance(Instance instance)`             Classifies a given instance after filtering. |
| `java.lang.String` | `filterTipText()`             Returns the tip text for this property. |
| `Capabilities` | `getCapabilities()`             Returns default capabilities of the clusterer. |
| `Filter` | `getFilter()`             Gets the filter used. |
| `java.lang.String[]` | `getOptions()`             Gets the current settings of the clusterer. |
| `java.lang.String` | `getRevision()`             Returns the revision string. |
| `java.lang.String` | `globalInfo()`             Returns a string describing this clusterer. |
| `java.util.Enumeration` | `listOptions()`             Returns an enumeration describing the available options. |
| `static void` | `main(java.lang.String[] args)`             Main method for testing this class. |
| `void` | `setFilter(Filter filter)`             Sets the filter. |
| `void` | `setOptions(java.lang.String[] options)`             Parses a given list of options. |
| `java.lang.String` | `toString()`             Output a representation of this clusterer. |

**Methods inherited from class weka.clusterers.SingleClustererEnhancer**

`clustererTipText, getClusterer, numberOfClusters, setClusterer`

**Methods inherited from class weka.clusterers.AbstractClusterer**

`clusterInstance, forName, makeCopies, makeCopy`

**Methods inherited from class java.lang.Object**

`equals, getClass, hashCode, notify, notifyAll, wait, wait, wait`

**Constructor Detail**

### FilteredClusterer

```
public FilteredClusterer()
```

:   Default constructor.


**Method Detail**

### globalInfo

```
public java.lang.String globalInfo()
```

:   Returns a string describing this clusterer.

    :   **Returns:**: a description of the clusterer suitable for displaying in the explorer/experimenter gui

---


### listOptions

```
public java.util.Enumeration listOptions()
```

:   Returns an enumeration describing the available options.

    :   **Specified by:**: `listOptions` in interface `OptionHandler` **Overrides:**: `listOptions` in class `SingleClustererEnhancer`
    :   **Returns:**: an enumeration of all the available options.

---


### setOptions

```
public void setOptions(java.lang.String[] options)
                throws java.lang.Exception
```

:   Parses a given list of options.


    Valid options are:

    ```
     -F <filter specification>
      Full class name of filter to use, followed
      by filter options.
      eg: "weka.filters.unsupervised.attribute.Remove -V -R 1,2"
     (default: weka.filters.AllFilter)
    ```

    ```
     -W
      Full name of base clusterer.
      (default: weka.clusterers.SimpleKMeans)
    ```

    ```
     Options specific to clusterer weka.clusterers.SimpleKMeans:
    ```

    ```
     -N <num>
      number of clusters.
      (default 2).
    ```

    ```
     -V
      Display std. deviations for centroids.
    ```

    ```
     -M
      Replace missing values with mean/mode.
    ```

    ```
     -S <num>
      Random number seed.
      (default 10)
    ```

    :   **Specified by:**: `setOptions` in interface `OptionHandler` **Overrides:**: `setOptions` in class `SingleClustererEnhancer`
    :   **Parameters:**: `options` - the list of options as an array of strings **Throws:**: `java.lang.Exception` - if an option is not supported

---


### getOptions

```
public java.lang.String[] getOptions()
```

:   Gets the current settings of the clusterer.

    :   **Specified by:**: `getOptions` in interface `OptionHandler` **Overrides:**: `getOptions` in class `SingleClustererEnhancer`
    :   **Returns:**: an array of strings suitable for passing to setOptions

---


### filterTipText

```
public java.lang.String filterTipText()
```

:   Returns the tip text for this property.

    :   **Returns:**: tip text for this property suitable for displaying in the explorer/experimenter gui

---


### setFilter

```
public void setFilter(Filter filter)
```

:   Sets the filter.

    :   **Parameters:**: `filter` - the filter with all options set.

---


### getFilter

```
public Filter getFilter()
```

:   Gets the filter used.

    :   **Returns:**: the filter

---


### getCapabilities

```
public Capabilities getCapabilities()
```

:   Returns default capabilities of the clusterer.

    :   **Specified by:**: `getCapabilities` in interface `Clusterer` **Specified by:**: `getCapabilities` in interface `CapabilitiesHandler` **Overrides:**: `getCapabilities` in class `SingleClustererEnhancer`
    :   **Returns:**: the capabilities of this clusterer **See Also:**: `Capabilities`

---


### buildClusterer

```
public void buildClusterer(Instances data)
                    throws java.lang.Exception
```

:   Build the clusterer on the filtered data.

    :   **Specified by:**: `buildClusterer` in interface `Clusterer` **Specified by:**: `buildClusterer` in class `AbstractClusterer`
    :   **Parameters:**: `data` - the training data **Throws:**: `java.lang.Exception` - if the clusterer could not be built successfully

---


### distributionForInstance

```
public double[] distributionForInstance(Instance instance)
                                 throws java.lang.Exception
```

:   Classifies a given instance after filtering.

    :   **Specified by:**: `distributionForInstance` in interface `Clusterer` **Overrides:**: `distributionForInstance` in class `AbstractClusterer`
    :   **Parameters:**: `instance` - the instance to be classified **Returns:**: the class distribution for the given instance **Throws:**: `java.lang.Exception` - if instance could not be classified successfully

---


### toString

```
public java.lang.String toString()
```

:   Output a representation of this clusterer.

    :   **Overrides:**: `toString` in class `java.lang.Object`
    :   **Returns:**: a representation of this clusterer

---


### getRevision

```
public java.lang.String getRevision()
```

:   Returns the revision string.

    :   **Specified by:**: `getRevision` in interface `RevisionHandler` **Overrides:**: `getRevision` in class `AbstractClusterer`
    :   **Returns:**: the revision

---


### main

```
public static void main(java.lang.String[] args)
```

:   Main method for testing this class.

    :   **Parameters:**: `args` - the commandline options, use "-h" for help


---


|  |  |  |
| --- | --- | --- |
| **Overview** \| **Package** \| **Class** \| **Tree** \| **Deprecated** \| **Index** \| **Help** \| **Weka's home** | |  |
| **PREV CLASS**   **NEXT CLASS** | **FRAMES**    **NO FRAMES**     **All Classes** |  |
| SUMMARY: NESTED \| FIELD \| CONSTR \| METHOD | DETAIL: FIELD \| CONSTR \| METHOD |  |


---
